# Factors Associated with Women's Medication Use

**DOI:** 10.1186/1472-6874-4-S1-S29

**Published:** 2004-08-25

**Authors:** Jennifer Payne, Ineke Neutel, Robert Cho, Marie DesMeules

**Affiliations:** 1 Centre for Chronic Disease Prevention and Control, Health Canada, 120 Colonnade Rd, Ottawa, Canada

## Abstract

**Health Issue:**

Research has consistently shown that while women generally live longer than men, they report more illness and use of health care services (including medication). In the literature, the reasons for women's elevated medication use are not clear. This paper investigates the associations between over-the-counter (OTC) and prescription (Rx) medication use and selected social and demographic variables in men and women.

**Key findings:**

While a larger proportion of women than men used medication throughout the study, the proportion of people using medication did not increase. The use of OTC and Rx medication increased by number of physician visits for women and men.

Medication use increased with age, chronic disease and number of physician visits, and decreased with the perception of good to excellent health. The relationship with other factors varied for women and men depending on their education level, income and social roles. For women, the social roles of being married or previously married, being employed or being a parent did not increase their likelihood of medication use. Reported income adequacy is not associated with the chances of mediation use among highly educated women, but for women with low levels, medication use increases as income adequacy decreases.

**Data Gaps and Recommendations:**

More complete data are needed about social roles and their relation to mediation use. Data that would allow an assessment of the appropriateness of OTC and Rx drug use or the reasons for such use need to be collected. More research is needed to better understand the distribution and determinants of specific medication use.

## Background

Researchers have shown that although women generally live longer than men,[1,2] they seem to report more illness more frequently [1,3-6] and use more health care services[1,3,4,7,8] and medication [9-14] than men. In Canada, the National Population Health Survey (NPHS) 1996–1997 found that a greater proportion of females aged 12 years and older were taking medication than males of the same age, and women were more likely than men to be taking one or two drugs but less likely to be taking three drugs at the same time.[1] Similarly, data from other countries, such as the United States,[15,16] the United Kingdom[17] and Australia,[18] have shown a higher use of medication and other health care services by women than by men. The reason for the excess use of health care services and medication is not clear, but several hypotheses have been suggested.

Women's high medication use may be associated with more physician visits. The evidence does suggest that women visit physicians more than men do [1,3,7,8] and use other diagnostic services (e.g. laboratory tests, blood pressure checks, prescriptions) more than men.[1,4,8,10] Further, women may be more likely to have a regular family physician,[1,4,7] which may make access easier. Some researchers have found that women are also more likely to make visits for non-symptomatic (preventive) reasons.[8]

Women are more likely to be given a prescription when visiting a physician. Verbrugge and Steiner, using the 1975 National Ambulatory Care data in the United States, studied drug prescribing for women and men who presented with the same complaints or medical diagnosis. They found that women received prescriptions more often than men, a difference that could not be explained by the medical differences in their conditions.[16] There is some evidence that patient expectation as well as physician characteristics, such as year of graduation and sex, may influence whether a prescription is given.[12,19] Mintzes and her colleagues[20] found that patients who requested a prescription were more likely to receive one (odds ratio [OR] 8.7, 95%, confidence interval [CI] 5.4, 14.2) than those who did not.

Medication use may be higher among women because women have a higher prevalence of chronic disease,[1,2,13] in particular a higher prevalence of painful conditions such as arthritis and musculoskeletal diseases.[14] In the NPHS 1996–1997, 18% of women reported having arthritis or rheumatism as compared with 10% of men.[1,3,21]

Since 16% of women of child-bearing age use birth control pills, and 11% of older women use hormonal therapy,[1,3] use of hormones could also be a reason for women's higher medication use.

Others have argued that women's increased use of health services may be linked to their social roles, particularly caregiving roles, which make them (i) more aware of and sensitive to health issues, (ii) more willing to seek professional help and (iii) more likely to follow the advice given.[4] In the literature, employment and marriage (having a partner) are associated with better health.[22] However, although non-partnered women who work and have children report poorer health than their peers without children, having children seems to make no difference for employed, married women.[4]

In general, prescription and medication use increase with age. Both the proportion of the population using medication and the number of medications used increases with age.[1,3] Women use more medications than men at all ages, from 20 years onwards.[23] However, the female-to-male (F/M) ratio decreases with age. Many researchers have shown that the F/M ratio for prescription drug use is highest in younger women and decreases in older age groups.[4,7]

This chapter looks at the extent to which social roles (being a parent, spouse, worker) and demographic and other risk factors are associated with medication use, categorized as prescription (Rx) and over-the-counter (OTC) medication use, in adults aged 20 years and older. For the main analyses hormone use was excluded as it is known that women have higher use of hormones.[1,3]

## Methods

### Data

Data from the NPHS 1994–1995, 1996–1997 and 1998–1999 were used to compare the proportion of women and men aged 20 years and over using "any medication," "over-the-counter medication" and "prescription medication." The data for this analysis are based on the question, "During the last 2 days how many drugs did you take?" This was asked only of those who said that they had taken medication in the preceding month. The data were weighted to reflect the Canadian population, and age-standardized to the 1991 Canadian population.

However, data from the 1996–1997 NPHS were used for most of the analyses because the effective sample of 61,879 allowed more precise estimates than other NPHS cycles (This sample size accounts for approximately 7,500 records in which the two-day detailed drug data were not coded.). The 1996–1997 NPHS, because of provincial buy-in, is larger than that of other NPHS cycles or of the drug module portion of the Canadian Community Health Survey (CCHS), a module chosen by only 29 of 136 regions, or 25% of the sample. Further, all of the regions choosing the CCHS drug module were from Ontario. For this more detailed analysis, data from the two-day drug recall component of the 1996–1997 NPHS (see Appendix A for details about methods of data collection) were used, in which respondents provided the names of drugs according to the container label. Neutel and Walop found this to be a more accurate reflection of drug use than responses to questions based on medication or disease categories[24]. A subsection of drugs were categorized according to whether they were OTC or Rx medication. (Drugs based on the ATC (Anatomical Therapeutic Chemical classification for medicinal products) codes for stomach problems, laxatives, pain/analgesics, coughs and colds, allergies, diabetes (insulin and oral hypoglycemics), cardiac conditions, hypertension (including diuretics), antibiotics, thyroid and asthma were categorized as Rx or OTC).

### Medication Use by Selected Variables

OTC and Rx medication usage by selected potential health determinants was compared between women and men and the F/M ratio calculated. In this part of the analysis, the roles of parent, partner and worker were considered. The role of parent and caregiver of children was assumed if the individual lived in a household with children under 6 years of age, and comparison was made with those who had no children under 6 years. Three categories of marital status were used, married/common law, previously married, and single or never married, the first two of which were compared with the last. With regard to working status, those who were currently working and had been doing so for the previous 12 months were compared with those who had not worked for the previous 12 months.

Other variables that were considered included geographic region; urban/rural location; educational level (involving two and four categories); income adequacy (this was based on the household income as well as the number of people in the household, and it was divided into four quartiles); immigrant status; reported experience of pain (four levels: no pain, mild, moderate and severe); social support (a five-point scale); physical activity (regular, occasional and infrequent); drug insurance availability; home ownership; perceived health (two- and five-category scales used); perceived stress (less than usual, usual and more than usual); presence of depression; presence of chronic diseases (none, one or two, and more than two); and number of physician visits (none or one, two to four, and more than five). These data were weighted to reflect the Canadian population and age-standardized to the 1991 Canadian population.

The pattern of medication use in individuals over 20 years of age was examined by number of physician visits (0–1, 2–4, and ≥ 5). This was done for OTC medications, prescription medications, and specific medications within these categories. Unlike the data presented in Figure 4, the data in Figure 5 were not age-standardized; however, the Figure provides specific categories of Rx and OTC medications for comparison.

### Multivariate Analysis

Multivariate analysis was used to examine the association between selected socio-demographic variables and Rx medication use alone as well as combined Rx and OTC medication use. The binary outcome variable was based on the use of one or more of the selected prescription medications in the previous two days. The analysis was limited to individuals between 20 and 54 years of age, as very few women and men 55 years or older had children under 6 years. Potential determinants included the social roles already described as well as education, income adequacy, number of chronic diseases, number of physician visits, perceived pain, perceived health (two levels), home ownership, drug insurance and age.

The modelling was done in three steps. In the first step, the full model was run, including interaction terms for sex and marital status, employment, parenthood, and education. All of the non-interaction terms were kept in the model as well as any interaction terms significant at the *p *< 0.05 level. There were significant interactions between sex and education, marital status and work. Because of the significant interaction with sex, for the second step men and women were modelled separately, in this case with interaction terms for education and marital status, employment, and parenthood. All of the non-interaction terms were kept in the model as well as any interaction terms significant at the *p *< 0.05 level. As there were significant education interaction terms for women and men, lower education (less than high school and high school level) and higher education (post-high school and degree) were modelled separately. Thus four separate multivariate models were run. The full model was run in each case for easier comparison. No attempt was made to find the best model. The data errors were estimated on the basis of 95% Wald confidence limits, determined by using the population weight rescaled to the sample size. While this procedure does not take into account the stratification or clustering of the sample design, it does improve the variance calculation, and it takes into account the unequal probabilities of selection.[25]

## Results

### Trends in Medication Use

**Figure 1:**
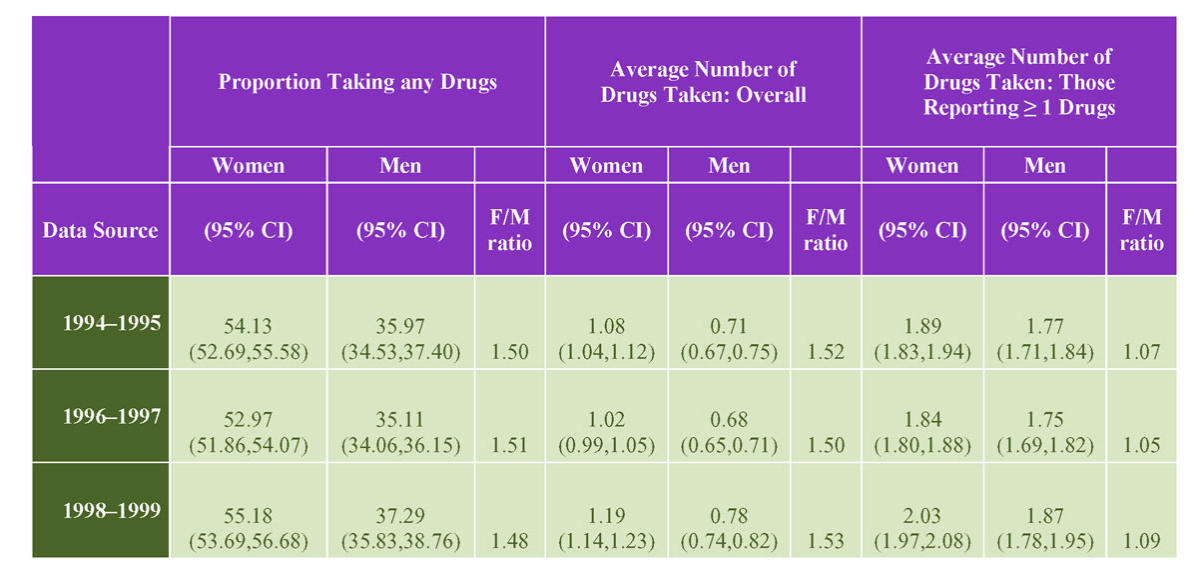
**Proportion of Canadians, 20 Years and Older, Using Any Medication and Average Number of Different Drugs Used. **Source: Statistics Canada, NPHS, various years. These data are based on "preceeding 2 days use" and are age standardized to the 1991 Canadian population

**Figure 2:**
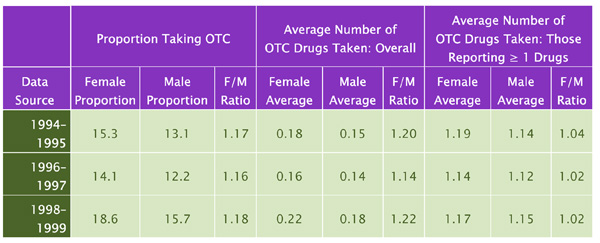
**Proportion of Canadians, 20+ Years Using Any Over the Counter (OTC) Medication and Average Number of Different Drugs Used **Source: Statistics Canada, NPHS, various years

**Figure 3:**
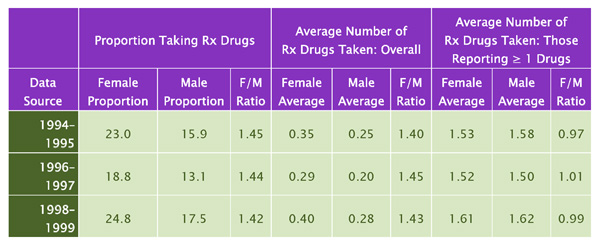
**Proportion of Canadians 20+ Years Using any Prescription (Rx) Medication and Average Number of Different Drugs Used **Source: Statistics Canada, NPHS, various years

Figure 1 shows the proportions of women and men 20 years and older who reported taking any medication and the average number of drugs being taken, both by those using *any *medication and those taking one drug or more, during the three NPHS periods under consideration. For each of the three periods, more women than men used medications. Further, there was no significant change in the proportion of men or women reporting use of medication over the three periods from 1994–1995 to 1998–1999. A subsection of medications, separated according to whether they were Rx or OTC, were similarly examined; these data are shown in Figures 2 and 3.

The F/M ratio for the average number of "any medications" taken and the average number of medications for those taking "one or more medications" were calculated. While the average number of "any medications" taken was higher for women, the F/M ratio is similar for those taking "one or more medications." The ratios were similar over the three periods. The OTC and Rx medications were examined separately and the F/M ratios calculated.

### Selected Determinants of Medication Use

**Figure 4:**
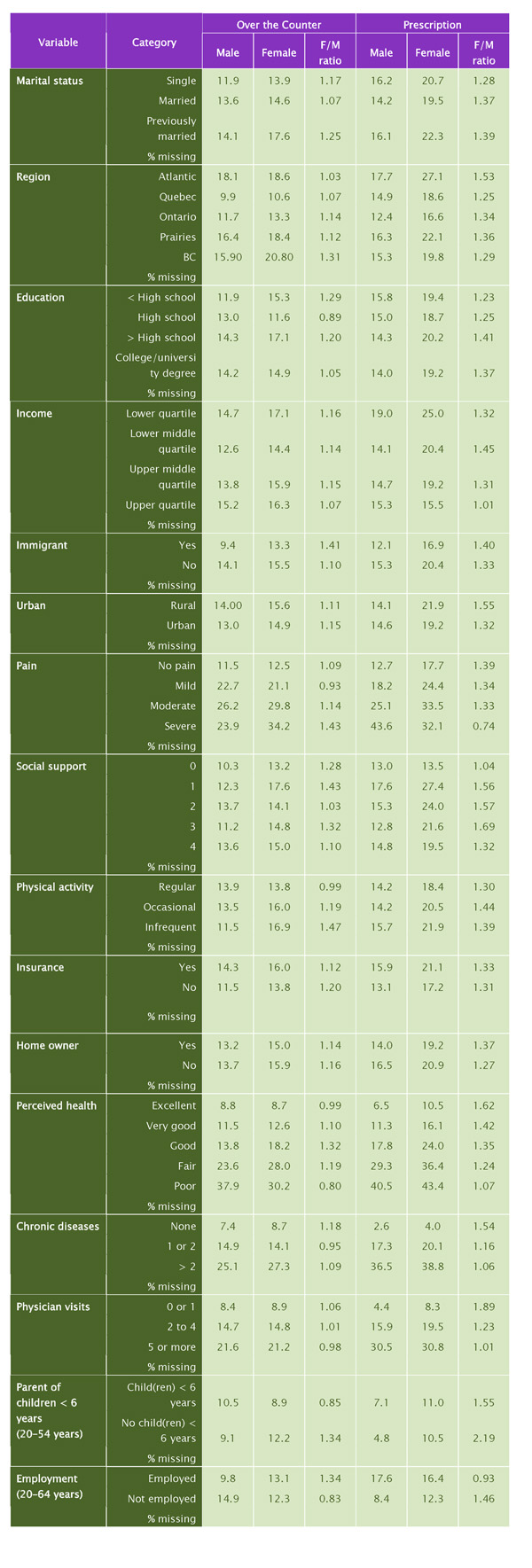
**Proportion of Canadians 20+ Years Taking Medications by Potential Determinants, Sex and Type of Medication (Age-Adjusted to 1991 Canadian Population) **Source: Statistics Canada, NPHS, 1996–1997

Figure 4 shows the age-standardized proportion of women and men and F/M ratios for OTC and Rx reported medication use by a variety of social and demographic factors. For both medication groups the estimated proportion of women is higher than that of men for most categories. As might be expected, the proportion of women and men reporting medication use increases with pain, number of chronic diseases, decreased perception of health, decreased income and increased number of physician visits. Immigrants are less likely to report medication use than non-immigrants.

The relation between reported medication use and variables such as geographic region, social support, physical activity and education is unclear. The data also suggest that the patterns of use may differ by medication group. The F/M ratio for the Rx medication group is generally higher than for the OTC group for most categories. Further, the F/M ratios are highest when a reduced likelihood of medication use might be expected; for instance, in the absence of chronic disease, in younger age groups, with fewer physician visits and when health is perceived to be excellent. The F/M ratio for prescription medication use among individuals experiencing "no pain" is 1.39 as compared with 0.74 among individuals who indicate that they experienced "severe" pain. Similarly, for the category "excellent perceived health" the ratio is 1.62 as compared with 1.07 for the category "poor perceived health."

### Pattern of Medication Use (OTC and Rx) by Physician Visits

**Figure 5:**
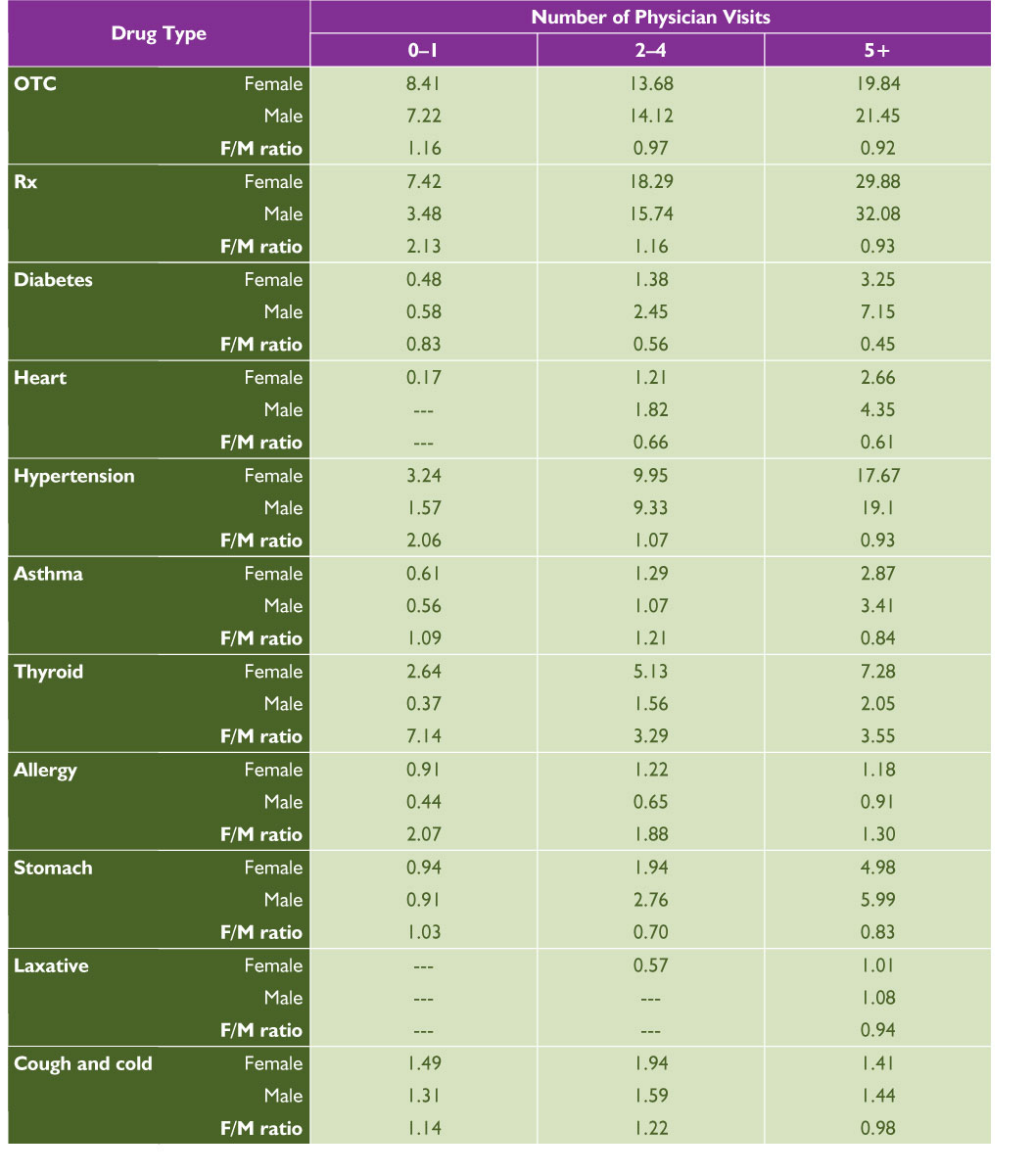
**Sex Specific Prevalence of Medication Use by Number of Physician Visits and Medication Type. (Not Age-Standardized) **Source: Statistics Canada, NPHS, 1996–1997

Figure 5 shows the proportion of women and men reporting OTC and Rx medication use by number of physician visits. As one would expect, medication use (OTC and Rx) increases among both men and women with number of physician visits. However, as shown in Figure 4, the F/M ratio is highest when the least medication use might be expected: it is highest in the 0–1 visit category and lowest for more than 5 visits. Further, this relation appears to be stronger for Rx medications than for OTC medications.

### Multivariate Analysis

**Figure 6:**
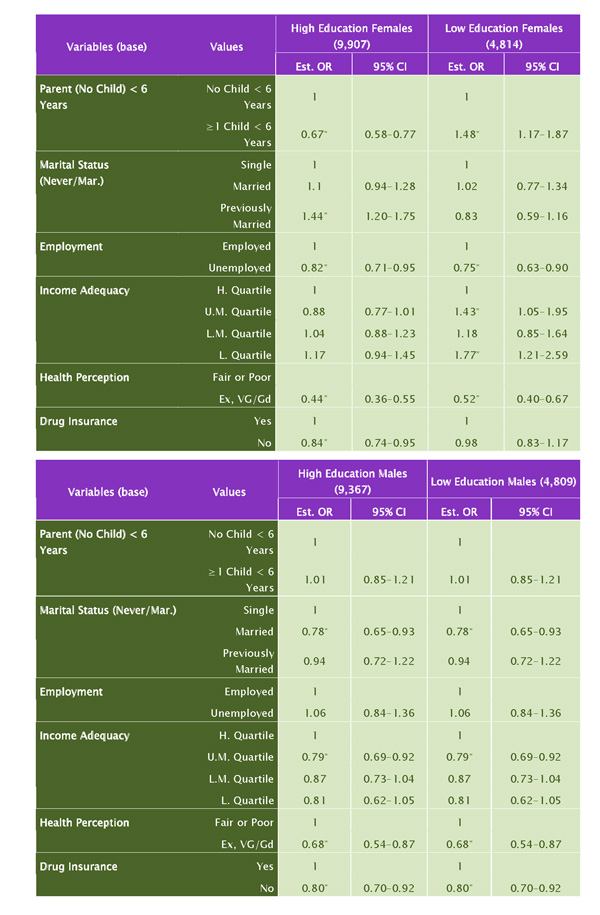
**Association Between Medication (Rx and OTC) Use and Selected Social and Health Factors: Results of Logistic Regression Analysis**. Source: NPHS 1996–1997 health share data (based on 2 day detailed data – Rx and OTC medication use). This sample accounts for approximately 7500 records in which the two-day detailed drug data was not coded.  "Drugs based on the ATC codes, for Stomach problems, laxatives, pain/analgesics, coughs and colds, allergies, diabetes (insulin and oral   hypo-glycaemics), cardiac conditions, hypertension (including diuretics), antibiotics, thyroid,   asthma were categorized as prescription or OTC."

**Figure 7:**
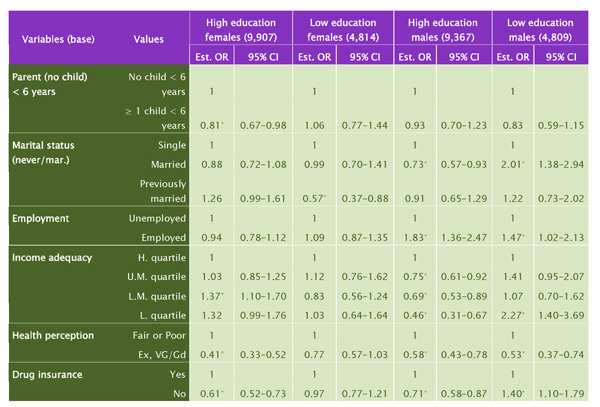
**Association Between Selected Medication (Rx) Use and Selected Social and Health Factors: Results of Logistic Regression Analysis **Source: NPHS 1996–1997 health share data (based on 2 day detailed data – Rx medication use). This sample accounts for approximately 7500 records in which the two-day detailed drug data was not coded.  "Drugs based on the ATC codes, for Stomach problems, laxatives, pain/analgesics, coughs and colds, allergies, diabetes (insulin and oral   hypo-glycaemics), cardiac conditions, hypertension (including diuretics), antibiotics, thyroid,   asthma were categorized as prescription or OTC."

Figures 6 and 7 present the results of the multivariate analyses of the association between the two medication use groups, Rx alone and Rx plus OTC, and a selected group of social and demographic variables.

### Marriage and Family

The probability of reported medication use varies by sex, family structure and category of medication used. In the adjusted models examining the relation between medication use and the selected variables, being married does not have an effect on women's medication use (for either the Rx and OTC or the Rx group). However, in the Rx and OTC group, for highly educated women the likelihood of reported medication use is greater among previously married women (OR 1.44, CI 1.20, 1.75) than their single counterparts, and in the Rx group women with a lower level of education who reported being previously married have a reduced probability of medication use (OR 0.57, CI 0.37, 0.88) than their single counterparts. For both medication use categories, being married reduces the chances of medication use among highly educated men and increases them among men with a low level of education as compared with their single counterparts.

The effect of having one or more children aged less than 6 years in the household is associated with a reduced likelihood of reported medication use among highly educated women in both medication use groups. The odds ratios are 0.81 (CI 0.67, 0.98) in the Rx group and 0.67 (CI 0.58, 0.77) in the Rx and OTC group. In the Rx and OTC analyses, women and men with a low level of education have a greater likelihood of medication use than their childless counterparts (OR 1.48, CI 1.17, 1.87, and OR 1.34, CI1.06, 1.70 respectively). Having one or more children less than 6 years of age in the household does not affect the chances of medication use among highly educated men.

### Employment and Income

In the Rx and OTC medication use category, the analyses suggested that being unemployed increases the likelihood of reported medication use among men with a low level of education as compared with their employed counterparts (OR 1.50, CI 1.12, 2.00), while it decreases the likelihood of medication use among high- and low-income women (OR 0.82, CI 0.71, 0.95, and OR 0.75, CI 0.63, 0.90 respectively). In the Rx only medication category, unemployment is associated with significant increases in the likelihood of medication use among men with high and low educational level (OR 1.83, CI 1.36, 2.47, and OR 1.47, CI 1.02, 2.13 respectively) but does not have an effect in women. When the unemployed and employed groups are analyzed separately (data not shown) there is no association between sex and medication use in the unemployed group, but in the employed group women were significantly more likely to use medication (OR 1.19, CI 1.07, 1.33).

In the Rx group, household income adequacy seems to be unrelated to the reported likelihood of medication use among women with a low educational level. Among highly educated women, lower income adequacy is associated with a higher chance of medication use. In the group of highly educated men the likelihood of medication use decreases with income adequacy, while among men with a low educational level and low income adequacy the likelihood is greater.

In the Rx and OTC group, reported income adequacy is not associated with the chances of medication use among highly educated women; among women with a low level of education the likelihood of medication use increases as income adequacy decreases. Among highly educated men there is a slight decrease in the likelihood of medication use as income adequacy decreases, whereas among men with a low educational level there is a slight increase in the chances of medication use with a reduction in income adequacy. On the other hand, not having drug insurance seems to significantly reduce the chances of reported medication use in both men and women with a high educational level and to increase the chances of medication use among men with low educational level in both medication use categories.

### Health-Related Variables

Perceived good health is associated with a reduced likelihood of reported medication use in all four analysis groups and in both medication use groups. As would be expected, reported medication use increases with greater numbers of chronic diseases, physician visits and age in all four groups.

## Discussion

Our data suggest that the proportion of individuals reporting medication use in each age group and the average number of medications being used per person remained steady between 1994–1995 and 1998–1999. Thus increased individual use of medication does not appear to have contributed to the rapidrise in spending for prescription drugs (totalling $12.3 billion in 2001–2002).[26]

More women than men in all categories report using medication, and these results are consistent with the literature.[4,27] However, for those taking one or more medications the F/M ratio is approximately 1, suggesting that for medication users the average number of drugs for males and females is similar. One of the most interesting findings in this study was the paradoxically higher F/M ratio for categories in which medication use might be expected to be the lowest. This higher F/M ratio for health care use in apparently low-risk situations has been reported elsewhere.[4] Verbrugge noted that the sex differences in health actions taken for life-threatening diseases were small, but by contrast the non-fatal, bothersome conditions were most likely to reveal sex differences.[4] This suggests that the excess medication use may be discretionary, as it seems to occur less in situations of obvious illness (such as when severe pain is present), when perceived health is poor or when five or more physician visits are required. In these data the pattern is more pronounced for Rx than for OTC medications. However, it is not clear whether the excess medication use reported by women represents appropriate self-care or the misuse/overuse of medication. Research is needed to better understand the paradoxically higher F/M ratio for probability of medication use in apparently low-risk categories.

### Marriage and Family

In these data, parenthood (defined as the presence of children under 6 years old in the household) has a different impact on reported medication use, depending on the type of medication used as well as educational level. Among women with high educational levels who have children there is a decreased likelihood of medication use (in both medication use groups), whereas among both women and men with low educational levels, having children in the household increases the reported medication use (Rx and OTC group). The reason for these differences is unclear. One possibility is that women and men with less education are less aware of health issues in general, but when there is a young child in the household the need to care for the child and the resulting increase in health knowledge may spill over and lead to an increased chance of Rx and OTC medication use. On the other hand, highly educated women may already be knowledgeable about their health and involved in self-care, but caring for a child may leave less time for self-care. Nooijer and colleagues found that being female and highly educated were positively associated with paying attention to cancer symptoms.[28]

In the fully adjusted data, being married has little effect on medication use. This is consistent with the findings of Bardel.[29] However, there is an effect for previously married women. The chances of medication use were reduced among women with a low level of education in the Rx group and were greater among highly educated women in the Rx and OTC group. This is not due to factors obviously related to marital status, such as access to a drug plan (controlled for) or the use of hormones (these were excluded from the analysis). Again, as we do not have any information about the appropriateness of the medication use, it is not possible to interpret these changes. For example, the increased likelihood of medication use among highly educated, previously married women could be due to increased awareness of health issues, preventive action and more time, or it could constitute inappropriate use of medication. It is important to have this information before recommendations can be made.

### Education, Employment and Income

As mentioned earlier, education is the socio-economic variable that seems to most affect the reported use of medication in these data analyses. This result is again consistent with that of Bardel et al.,[29] who also found education to be a significant predictor of medication use.

These results do not support the hypothesis that employment is protective against medication use among women. Women with high and low educational levels are more likely (in the Rx and OTC group) to use medication when employed (unlike men). Further, the lack of a sex difference in non-worker medication use suggests that unemployed (stay-at-home) men may have a similar pattern of medication use to that of women.

There is not a consistent relation between income adequacy and medication use in these data. However, the data suggest that (depending on the type of medication used) the chances of medication use may increase or stay the same as income adequacy decreases, for women and men of low educational level. On the other hand, they suggest that for highly educated men, as income adequacy decreases the likelihood of medication use also decreases.

Here again more research is needed to better understand how these variables are connected.

### Data and Knowledge Gaps

The analysis in this chapter provides some insight into gender-related patterns of medication use. However, a better understanding of the determinants and appropriateness of women's medication use could be gained if data were available on the roles that women have, the quality of their roles and the reasons for medication use. Currently, some of this information is available, but it is in different databases and difficult to link.

## Recommendations

• Data collection should include information on the quality and duration of social roles, as research suggests that these have a considerable effect on health impact. [30-32] For example, for the partnership role, data were available on marital status but not on the woman's perception of the quality of the relationship, or on same-sex relationships or the duration of a relationship. For parenting, the amount of time spent on child care or housework and an assessment of the quality of the role are lacking. Similarly, although employment is generally "healthy," its health-promoting effects are conditional on the type of work involved,[30] and this information is not available.

• NPHS cross-sectional data were used; therefore, it was impossible to capture the temporal relations between associated variables. Longitudinal data are necessary to examine these relations; while NPHS longitudinal data are available, important contextual variables are not available. As medication cost is the fastest growing part of health care costs,[18] the data required for an examination and understanding of the factors involved in medication use are important and should be monitored.

• Being able to assess the appropriateness of drug use is important, and thus it would be helpful to collect data on the reasons why medications are used as well as which ones are used. Savoie and Kazanjian, looking at the appropriateness of statin (lipid-lowering drugs) prescriptions, found that 88.7% of the utilization in their study was inconsistent with evidence for statin use in theliterature.[33]

• According to the NPHS 1996–1997, almost two thirds of the Canadian population aged 12 years and over were taking some form of medication, and 30% overall were taking three or more medications at the same time.[1] In a B.C. drug study, 48% of the individuals exposed to six or more different drugs received prescriptions from three or more different physicians. [34] It is important to have a system in place that can adequately monitor and provide feedback to reduce drug interactions.

• More research is needed about how ethnicity and the circumstances of different ethnic groups affect their access to and use of the health care system, including medications.

• Research is needed to better understand the apparent excess risk of medication use by women in circumstances under which medication use might be expected to be lowest.

## Notes

The authors wish to acknowledge the contribution of Rose Odili, BPharm, who separated the selected group of drugs used in the analysis into prescription and over-the-counter categories. The views expressed in this report do not necessarily represent the views of the Canadian Population Health Initiative, the Canadian Institute for Health Information or Health Canada.
